# Effects of eight-week resistance and complex training on the biomechanical characteristics of lower limbs during badminton backhand forward lunge

**DOI:** 10.3389/fspor.2025.1615558

**Published:** 2025-06-03

**Authors:** Zhonghao Xie, Xingyu Wu, Huiting Liang, Wei Li, Renjie Li, Zhe Wang, Tao Wen, Zhiguan Huang

**Affiliations:** ^1^ Guaduate School, Guangzhou Sport University, Guangzhou, China; ^2^ Ersha Sports Training Center of Guangdong Province, Guangzhou, China; ^3^ Guangdong Engineering Technology Research Center for Sports Aids, Guangzhou, China; ^4^ School of Sports and Health, Guangzhou Sport University, Guangzhou, China

**Keywords:** badminton, lunge, resistance training, complex training, lower limb biomechanics

## Abstract

**Objectives:**

This study aimed to examine the effects of an 8-week resistance training (RT) or complex training (CT) program on lower limb biomechanical characteristics during the backhand forward lunge in male amateur badminton players.

**Methods:**

Twenty male amateur badminton players were randomly assigned to either a complex training group or a resistance training group for an eight-week intervention. Lower limb kinematics and dynamics were captured before and after the intervention using an eight-camera Vicon motion system and two AMTI force plates. The measured variables included phase time, joint angles, joint range of motion (ROM), and joint moments during the stance phase. Discrete variables were analyzed using Generalized Estimating Equations (GEE), while continuous variables were evaluated using Statistical Parametric Mapping 1D (SPM1D).

**Results:**

The RT group significantly reduced recovery phase time (0.3 vs. 0.28 s, *p* < 0.01) and increased ankle transverse plane ROM (16.83° vs. 20.61°, *p* = 0.005), along with improvements in hip and knee flexion angles and ankle plantarflexion angle. The CT group significantly reduced both braking phase time (0.32 vs. 0.28 s, *p* = 0.002) and recovery phase time (0.31 vs. 0.28 s, *p* < 0.01), as well as decreased knee sagittal ROM (83.30° vs. 78.84°, *p* = 0.04).

**Conclusion:**

Both training interventions enhanced the performance of the backhand forward lunge. Complex training resulted in greater improvements in execution efficiency, while resistance training not only improved efficiency but also demonstrated potential for reducing knee and ankle injury risk.

## Introduction

Badminton is one of the most popular sports in the world, with 200 million adherents (1). Players frequently perform strenuous movements such as lunging, turning, sprinting, leaping, jumping, and landing, all of which are critical for successful gameplay (2). Among these movements, effective footwork is fundamental, as it enables players to position themselves optimally for shots and quickly return to the base position in preparation for their opponents’ returns (3, 4). Among various footwork types, lunges are particularly prevalent, accounting for over 15% of all movements during gameplay (2, 5). Players could experience an impact load as high as 2.5 times the body weight and require sufficiently high muscle activities to stabilize lower extremity joints during a lunge (1). However, the demanding footwork could also result in high injury risks of the lower limb (6), making it essential to understand the underlying biomechanical characteristics and ways to mitigate these risks.

Lunges in badminton can be categorized into four primary directions: right-forward, left-forward, right-backward, and left-backward steps (5). Among these, the backhand (left/non-racket side) forward lunge is considered the most critical, as it produces the highest plantar loading compared to other lunge directions (5). Additionally, this movement requires greater trunk rotation, core stability, and dynamic postural control than forehand forward lunges (7). Given these biomechanical demands and the increased risk of lower limb stress, it is essential to further investigate the backhand forward lunge to optimize performance and develop injury prevention strategies.

Sports performance strongly relies on the capacity to generate maximal neuromuscular power (8), which requires both sufficient strength and the effective transfer of that strength to dynamic movements (9). Resistance training (RT) is a typical training approach to enhance the maximal strength and power in athletes (10). RT can be defined as the ability of a given muscle or group of muscles to generate muscular force under specific conditions (11). Its physiological benefits include increases in phosphagen stores, contractile proteins, anaerobic power output, muscle architecture, fibre pennation, and protein synthesis (11–13). Incorporating RT into training programs may help badminton players perform lunges more efficiently. However, the specific effects of RT on lower limb biomechanics during badminton lunges remain unclear. Investigating these effects could offer valuable insights for optimizing badminton training strategies.

In addition to RT, complex training (CT) integrates plyometrics with similar biomechanical characteristics immediately following resistance training within a single session (14, 15). Many researchers regard CT as a safer and potentially more effective approach to strength and power development than isolated training methods (16, 17). Studies indicate that CT interventions typically require at least three weeks, with a training frequency of 2–4 sessions per week, to elicit adaptations in various physical performance parameters (15, 18–20). However, there is limited research on the effects of these training interventions on badminton lunges. Additionally, whether RT and CT are equally effective in enhancing backhand forward lunge performance remains unclear. Investigating their respective impacts could provide important guidance for selecting optimal training strategies.

Therefore, we recruited male amateur badminton players and randomly assigned them to either a resistance training group or a complex training group. The purpose of this study was to examine the effects of an 8-week resistance training and complex training program on the biomechanical characteristics of the backhand forward lunge. We hypothesized that both training interventions would enhance participants’ backhand forward lunge performance, with complex training leading to greater improvements.

## Methods

### Experimental design

**Figure 1:**
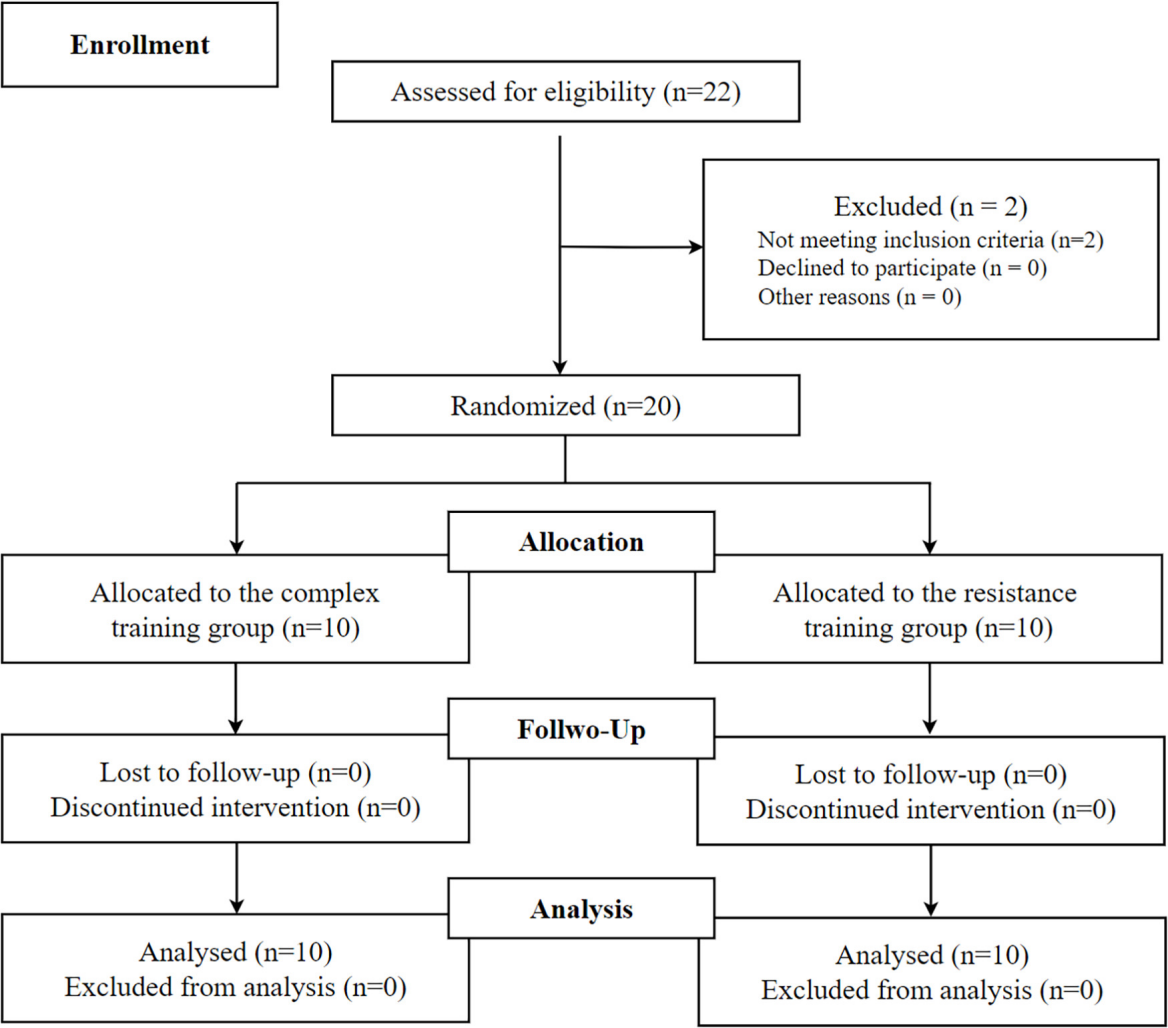
Flow chart of the progress through the phases of the study according to the CONSORT statements.

The study was conducted on amateur male badminton players using a randomized parallel design structured into a 10-week macrocycle, consisting of a one-week pre-test phase, an eight-week training intervention, and a one-week post-test phase (Figure 1). Participants were randomly assigned to either the Complex Training (CT) group or the Resistance Training (RT) group. In the first week, all players attended a familiarization session (Monday) to ensure they understood all testing and training procedures. During this session, their standing height and body mass were measured. Forty-eight hours later (Wednesday), the participants completed a biomechanical pre-test of the backhand forward lunge. On Friday, they underwent a one-repetition maximum (1RM) test to determine baseline strength levels. Following the 16-session training period, the participants completed biomechanical post-tests of the backhand forward lunge, administered in the same manner as the pre-test.

### Participants

Twenty amateur male badminton players from Guangzhou Sport University were recruited and randomly assigned to one of two groups: the Complex Training (CT) group (*n* = 10; height = 1.75 ± 0.07 m; body weight = 62.25 ± 9.01 kg; BMI = 19.93 ± 1.71 kg/m²) or the Resistance Training (RT) group (*n* = 10; height = 1.73 ± 0.07 m; body weight = 60.07 ± 9.07 kg; BMI = 20.1 ± 1.79 kg/m²). Randomization was performed using an online randomization tool (https://www.random.org). The sample size was determined based on previous similar research studies (21).

The inclusion criteria were as follows: (1) engaged in regular badminton practice (at least six hours per week); (2) a minimum of two years of playing experience; (3) aged between 18 and 24 years; (4) right-handedness; (5) no history of lower limb injury in the past year; (6) no prior plyometric or resistance training experience in the past six months. Ethical approval was obtained from the Ethics Committee of Guangzhou Sport University (Approval No. 2022LCLL-12). The experiments were performed in accordance with the ethical standards of the Helsinki Declaration, and all participants signed an informed consent form.

### Experimental procedures

#### Backhand forward lunge test

**Figure 2:**
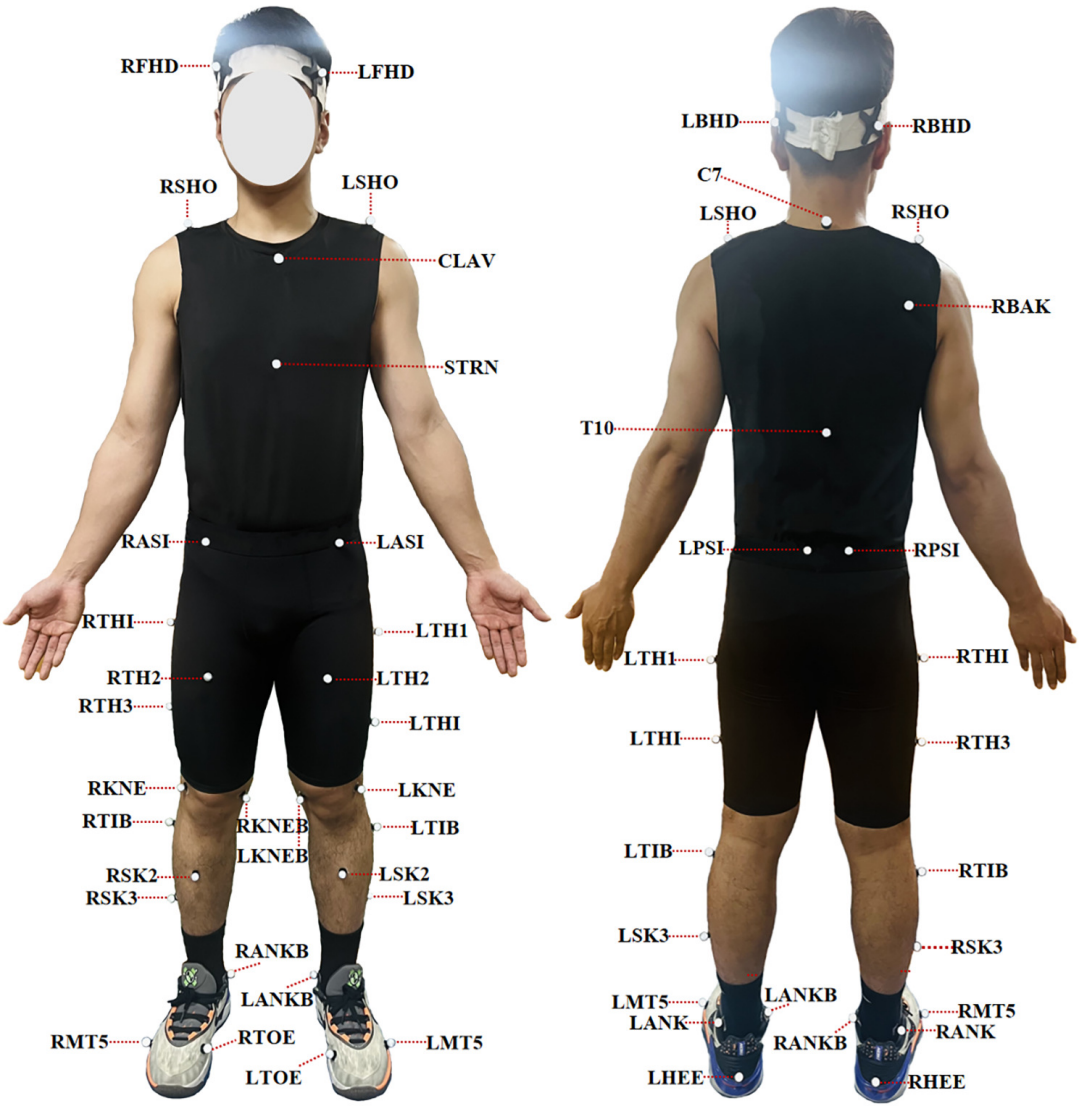
Marker placement. The marker placement locations included the following anatomical landmarks: the brow bone, occipital bone, acromion, C7 vertebra, center of the right scapula, T10 vertebra, center of the clavicle, lowest point of the sternum, anterior and posterior superior iliac spines, medial and lateral femoral condyles, lateral thigh, lateral calf, medial and lateral malleoli, heel, and the first and fifth metatarsal heads.

The pre-test and post-test for the backhand forward lunge were conducted at the Guangdong Sports Equipment Engineering Technology Research Center. Each participant performed a three-step lunge technique. An eight-camera Vicon motion capture system (Oxford Metrics Ltd., Oxford, UK), sampling at 200 Hz, was utilized to collect raw kinematic data during the badminton lunge. A reflective marker set consisting of 43 markers (diameter: 14 mm) was attached to the participants’ bodies to define joint segments and axes of rotation (see Figure 2). Raw dynamic data were recorded using two AMTI force platforms (Advanced Mechanical Technology Inc., Watertown, MA, USA), operating at a sampling frequency of 1,000 Hz. Kinematic and dynamic data were collected simultaneously.

**Figure 3:**
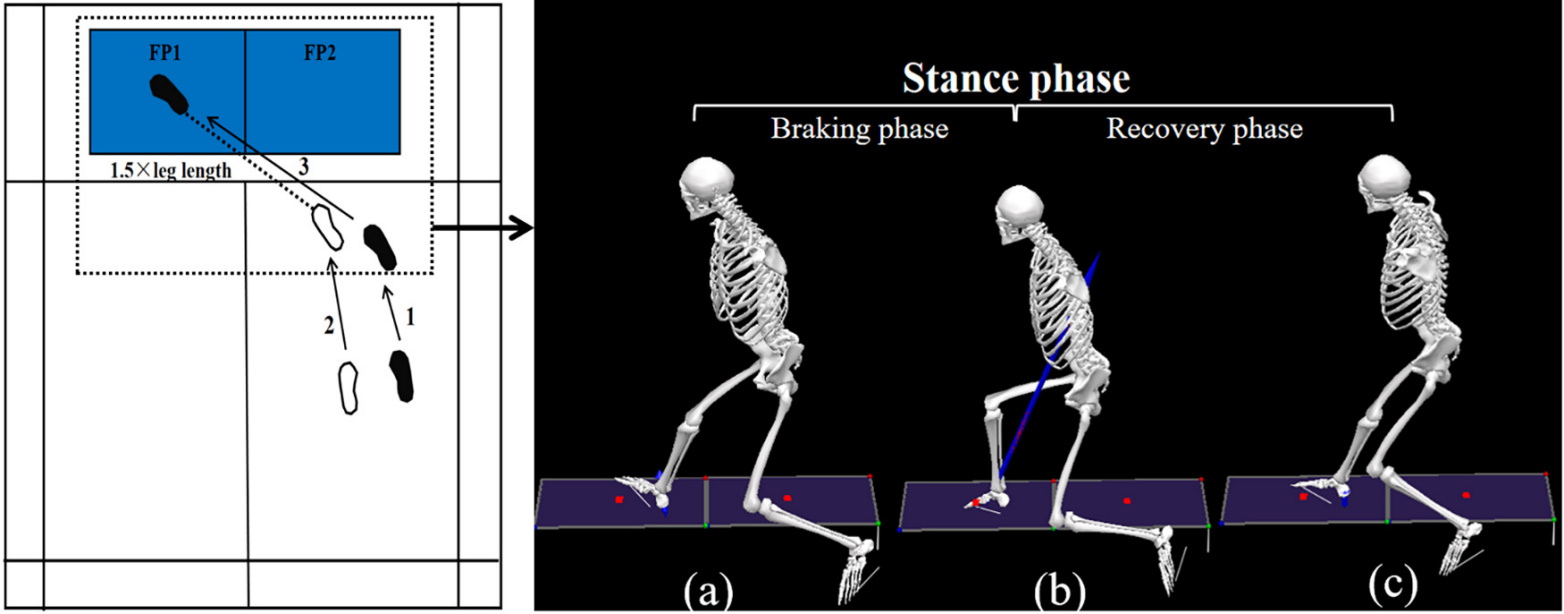
Schematic diagram of backhand forward lunges. White footprints indicate the position of the left foot, and black footprints indicate the position of the right foot, with numbers indicating the step sequence. FP1 and FP2 represent two different force plates. The dashed line between the footprints represents 1.5 times the leg length. **(a)** Initial contact: the heel contacts the force plate, with vertical ground reaction force >15 N; **(b)** Moment of minimum flexion angle: the point at which the dominant knee joint achieves its minimum flexion angle during the lunge; **(c)** Lift-off: the dominant leg leaves the force plate, with vertical ground reaction force <15 N. The period from **(a,b)** indicates the braking phase, **(b,c)** indicates the recovery phase, and **(a–c)** indicates the stance phase.

Test Preparation Phase: To standardize lunge distance (2, 22), the starting position for each participant was set at 1.5 times their individual leg length (measured from the anterior superior iliac spine to the lateral malleolus) and positioned at a 45-degree angle relative to the *x*-axis of the force platforms. The designated endpoint was centered on the force plate to ensure uniform landing positions (see Figure 3). Participants completed a 10-minute standardized warm-up, including dynamic stretching and practice lunges.

Formal Testing Phase: Upon receiving the start command, participants held a badminton racket and initiated the backhand forward lunge from the designated starting position. They were instructed to execute the lunge with maximum effort, simulating competition-like conditions. Participants were required to ensure that the racket-holding leg landed within the designated endpoint before returning to the starting position as quickly as possible. Each participant completed five valid trials of the backhand forward lunge. A 30-second rest interval was provided between trials. A trial was considered valid if the participant's front foot landed within the boundaries of the force platform, and no noticeable slippage occurred.

#### One repetition maximum back squat testing

The 1RM test measures the maximum load an individual can lift through a controlled squat movement, reaching 90° knee flexion before returning to a fully upright position. Initially, participants performed a warm-up with 5–6 repetitions at a light load. This was followed by 3–4 repetitions at a moderate load to prepare the muscles for maximum effort. Then, a single repetition at ∼95% of the estimated 1RM was performed to assess readiness for the actual 1RM attempt. Participants attempted to lift a load perceived as their maximum capacity. If successful, the weight was incrementally increased by 1.0–2.5 kg for additional attempts. A 2-minute rest interval was provided between trials to minimize fatigue and ensure accurate assessment. A test attempt was considered unsuccessful if the participant failed to complete the squat through the full range of motion (90° knee flexion) in at least two consecutive attempts. Typically, 4–5 trials were required to precisely determine the 1RM (23).

#### Training program

Both the Complex Training (CT) group and the Resistance Training (RT) group completed an 8-week intervention, consisting of two training sessions per week, with each session spaced 72 hours apart. The training program was structured into four progressive phases, each lasting two weeks, and was conducted at the Physical Training Center of Guangzhou Sport University.

**Figure 4:**
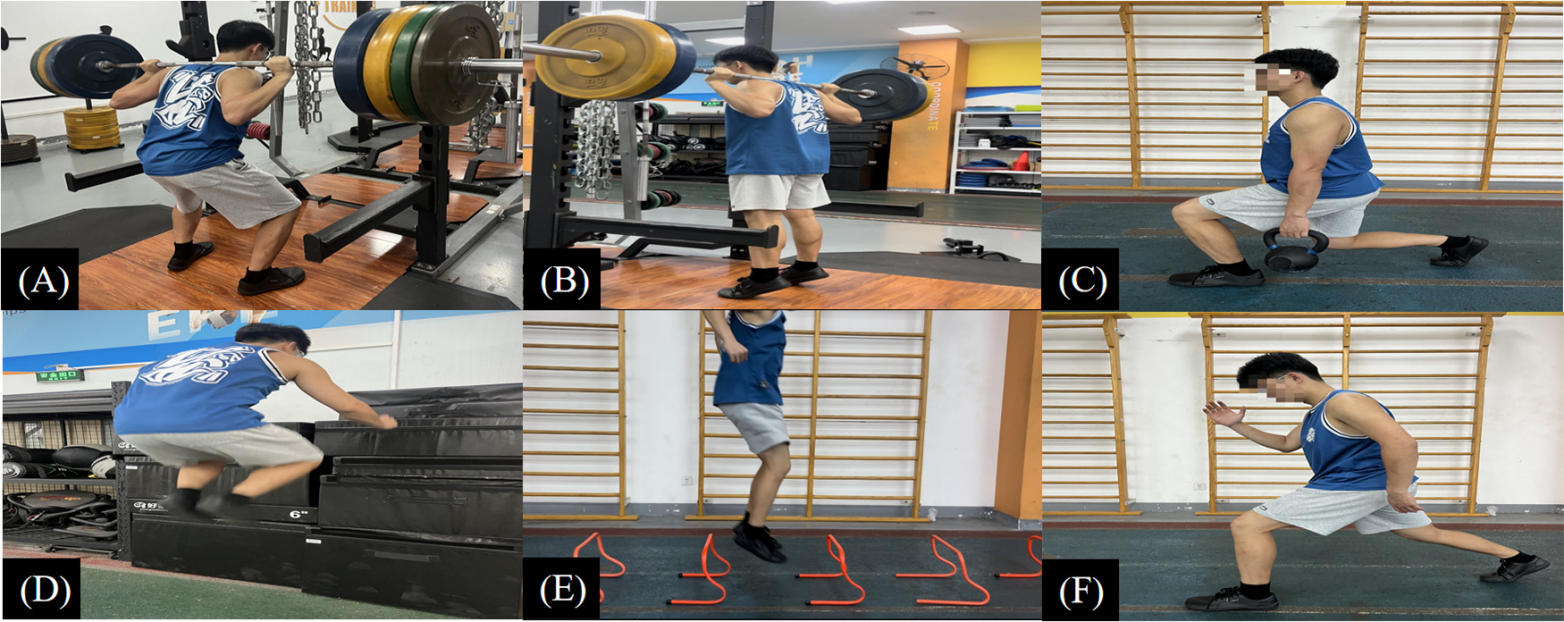
Schematic diagram of the training exercises. **(A)** Squat, **(B)** calf raise, **(C)** split squat, **(D)** vertical jump, **(E)** hurdle jump, **(F)** jumping lunge. The resistance training program exercises included **(A–C)**. The complex training program exercises included **(A)** paired with **(D,B)** paired with **(E,C)** paired with **(F)**.

Complex Training (CT) Protocol: The CT program exercise combinations included: (1) squats paired with vertical jumps; (2) calf raises paired with hurdle jumps; and (3) split squats paired with jumping lunges (see Figure 4). Each exercise combination was performed for three sets per session, with resistance exercises (squats, calf raises, split squats) executed four repetitions per set, and plyometric exercises (vertical jumps, hurdle jumps, jumping lunges) executed eight repetitions per set. A 30-second rest was provided between paired exercises within a set, and a three-minute rest was given between sets. The training load intensity was progressively increased across phases (see Table 1).

**Table 1:** Complex training and resistance training program.

Exercise	Weeks 1–2	Weeks 3–4	Weeks 5–6	Weeks 7–8	Repetitions	Set	RestSet
Complex training							
Squat + vertical Jump	75% 1RM + BW	80% 1RM + BW	85% 1RM + BW	90% 1RM + BW	4 + 8	3	3 min
Calf raise + hurdle jumps	50% 1RM + BW	55% 1RM + BW	60% 1RM + BW	65% 1RM + BW			
Split squat + jumping lunge	20 KG + BW	25 KG + BW	30 KG + BW	35 KG + BW			
Resistance training							
Squat	75% 1RM	80% 1RM	85% 1RM	90% 1RM	6	3	3 min
Calf raise	50% 1RM	55% 1RM	60% 1RM	65% 1RM			
Split squat	20 KG	25 KG	30 KG	35 KG			

%1RM, percentage of one-repetition maximum load intensity; RM, maximum repetitions; BW, body weight.

Resistance Training (RT) Protocol: The RT program exercises included: (1) squats; (2) calf raises; and (3) split squats (see Figure 4). Each exercise was performed for three sets per session. Each exercise was executed for six repetitions per set. A three-minute rest was provided between sets. Similar to the CT group, the training intensity was gradually increased throughout the intervention (see Table 1).

### Data reduction and analysis

Spline interpolation was performed to fill minor missing marker trajectories in the Vicon Nexus 2.15.0 software (Oxford Metrics Ltd., Oxford, UK). All kinematic and dynamic data were subsequently imported into Visual3D software (C-Motion Inc., Rockville, MD, USA) for further processing. Kinematic and dynamic data were filtered using a fourth-order Butterworth filter with cutoff frequencies of 15 and 25 Hz, respectively (24).

The following variables were analyzed: kinematic variables, which included joint angles and joint range of motion (ROM). The three-dimensional kinematics of the joints was calculated using an XYZ Cardan sequence of rotations (X: flexion-extension, Y: abduction-adduction, and Z: internal-external rotation). ROM was defined as the difference between the maximum and minimum joint angles of the hip, knee, and ankle joints during the stance phase. Dynamic variables included joint moment. Net joint moment was calculated using the Newton-Euler inverse dynamics approach. A positive value for joint angle and moment denoted hip flexion, adduction, and internal rotation; knee flexion, varus, and internal rotation; ankle dorsiflexion, inversion, and adduction for respective orthogonal planes.

The variables were computed during the stance phase. The stance phase was defined as the period from initial contact to final lift-off of the racket-holding leg from the force plate. Contact and lift-off events were identified based on the vertical reaction force, with a cutoff threshold of 15 N (2). Moreover, the stance phase was further divided into a braking phase and a recovery phase (Figure 3). Joint angle and joint moment time series data were normalized to 101 frames. All kinetic data were normalized to each participant's body weight (BW). The mean of three valid trials for each variable was then used for the analysis.

### Statistical analysis

We compared phase time, joint range of motion (ROM) between groups and time points using generalized estimating equations (GEE), with time and group as factors, and applied baseline covariate adjustment. When necessary, Bonferroni *post hoc* tests were conducted to identify significant differences. Given the one-dimensional (1D) nature of the joint angle and joint moment data, Statistical Parametric Mapping 1D (SPM1D) was applied to analyze motion across the three planes of motion (3). Paired-sample t-tests within SPM1D (http://www.spm1d.org/index.html) were used to assess pre-training and post-training differences within each training group.

To quantify the effect sizes (ESs) of the mean differences between pre-training and post-training moments in the CT and RT groups, we calculated Hedges'g. The interpretation of ES magnitude was based on Hopkins’ criteria: <0.2 was trivial, 0.2–0.59 was small, 0.6–1.19 was moderate, 1.2–1.99 was large, and >2.0 was very large (21). All statistical analyses were conducted using SPSS version 17.0 (IBM, Armonk, NY, USA) and Python version 3.12.1 (Python Software Foundation, Wilmington, DE, USA). The significance level was set at *p* < 0.05.

## Results

A significant interaction effect between time and group was observed for braking phase time (*p* < 0.001). *post hoc* analysis showed that pre-training braking phase time was significantly shorter in the RT group than in the CT group (0.27 ± 0.01 vs. 0.32 ± 0.01 s, *p* = 0.002). Following training, braking phase time in the CT group was significantly reduced compared to its pre-training value (0.28 ± 0.01 vs. 0.32 ± 0.01 s, *p* < 0.001). Regarding recovery phase time, a significant decrease was observed from pre- to post-training across both groups (0.28 ± 0.01 vs. 0.31 ± 0.01 s, *p* < 0.001), independent of group assignment. No significant changes were detected for stance phase time. Effect size analysis for the CT group revealed trivial effects for both braking and stance phase time, and a small effect for recovery phase time. In the RT group, small effects were observed for braking and stance phase time, while a moderate effect was noted for recovery phase time (g = 1.02; 95% CI: 0.25–1.75) (Table 2).

**Table 2:** Effect of training intervention on the phase time.

Group	Pre-training (Mean ± SE)	Post-training (Mean ± SE)	Time (p)	Group (p)	Interaction (p)
Braking phase time (s)					
Complex training	0.32 ± 0.01	0.28 ± 0.01	0.19	0.98	<0.001
Resistance training	0.27 ± 0.01	0.28 ± 0.01			
Recovery phase time (s)					
Complex training	0.3 ± 0.01	0.28 ± 0.01	<0.001	0.67	0.26
Resistance training	0.31 ± 0.01	0.28 ± 0.06			
Stance phase time (s)					
Complex training	0.62 ± 0.02	0.56 ± 0.02	0.31	0.82	0.17
Resistance training	0.58 ± 0.01	0.56 ± 0.02			

For hip range of motion (ROM), no significant differences were found in the sagittal, coronal, or transverse planes. Effect size analysis for the CT group revealed trivial effects in coronal plane ROM, a small effect in sagittal plane ROM (g = 0.57; 95% CI: 0.09–1.20), and a moderate effect in transverse plane ROM (g = 0.73; 95% CI: 0.40–1.40). The RT group showed trivial effects in coronal and transverse plane ROM, and a small effect in sagittal plane ROM (g = 0.40; 95% CI: 0.20–1.01) (Table 3).

**Table 3:** Effect of training intervention on the joint range of motion.

Group	Pre-training (Mean ± SE)	Post-training (Mean ± SE)	Time (p)	Group (p)	Interaction (p)
Hip sagittal ROM (°)					
Complex training	77.67 ± 3.56	63.16 ± 2.64	0.09	0.16	0.88
Resistance training	82.86 ± 7.37	69.84 ± 2.93			
Hip coronal ROM (°)					
Complex training	19.59 ± 1.12	20.3 ± 1.61	0.86	0.52	0.68
Resistance training	22.25 ± 1.12	21.86 ± 1.84			
Hip transverse ROM (°)					
Complex training	49.19 ± 4.08	36.61 ± 3.45	0.58	0.61	0.29
Resistance training	43.59 ± 3.32	40.04 ± 5.18			
Knee sagittal ROM (°)					
Complex training	83.3 ± 2.68	78.84 ± 2.79	0.04	0.86	0.87
Resistance training	83.18 ± 1.77	78.26 ± 1.96			
Knee coronal ROM (°)					
Complex training	28.29 ± 2.68	23.36 ± 1.95	0.35	0.61	0.79
Resistance training	25.64 ± 2.72	21.89 ± 2.12			
Knee transverse ROM (°)					
Complex training	36.06 ± 2.1	31.9 ± 2.13	0.49	0.83	0.2
Resistance training	29.32 ± 2.06	32.71 ± 3.15			
Ankle sagittal ROM (°)					
Complex training	57.76 ± 2.54	52.58 ± 3.56	0.17	0.72	0.48
Resistance training	48.51 ± 3.96	46.88 ± 2.2			
Ankle coronal ROM (°)					
Complex training	14.38 ± 1.01	14.67 ± 1.75	0.62	0.11	0.78
Resistance training	19.11 ± 2.07	20.11 ± 2.93			
Ankle transverse ROM (°)					
Complex training	19.17 ± 1.46	18.61 ± 1.61	0.005	0.43	0.12
Resistance training	16.83 ± 1.61	20.61 ± 1.96			

For knee ROM, a significant reduction in sagittal plane ROM was observed from pre- to post-training (*p* = 0.04). *post hoc* analysis indicated that sagittal plane knee ROM in the CT group was significantly greater pre-training than post-training (83.3 ± 2.68° vs. 78.84 ± 2.79°, *p* = 0.02). Effect size analysis showed trivial effects in coronal plane ROM and moderate effects in both sagittal (g = 0.63; 95% CI: 0.07–1.29) and transverse plane ROM (g = 0.73; 95% CI: 0.40–1.40) for the CT group. The RT group showed trivial effects in coronal and transverse plane ROM, and a moderate effect in sagittal plane ROM (g = 0.62; 95% CI: 0.08–1.28).

For ankle ROM, a significant increase in transverse plane ROM was found in the RT group from pre- to post-training (20.61 ± 1.96° vs. 16.83 ± 1.61°, *p* = 0.005). Effect size analysis for the CT group revealed trivial effects across sagittal, coronal, and transverse planes. The RT group showed trivial effects in sagittal plane ROM, a small effect in coronal plane ROM, and a moderate effect in transverse plane ROM (g = 0.81; 95% CI: 0.10–1.50).

For the joint angle, SPM1D analysis revealed no significant differences in hip, knee, and ankle angles before and after training in the CT group. However, in the RT group, significant differences were observed in hip, knee, and ankle sagittal plane angles. Specifically, the sagittal plane hip angle exceeded the critical threshold of 4.01 during the 79%–87% phase (*p* = 0.009), indicating a significantly higher hip flexion angle post-training. The sagittal plane knee angle exceeded the critical threshold of 3.87 during the 98%–100% phase (*p* = 0.049), indicating a significantly higher knee flexion angle in the post-training. The sagittal plane ankle angle exceeded the critical threshold of 4.16 during the 13%–21% (*p* = 0.007) and 88%–92% phase (*p* = 0.03), indicating a significantly higher ankle plantarflexion angle pre-training.

**Figure 5:**
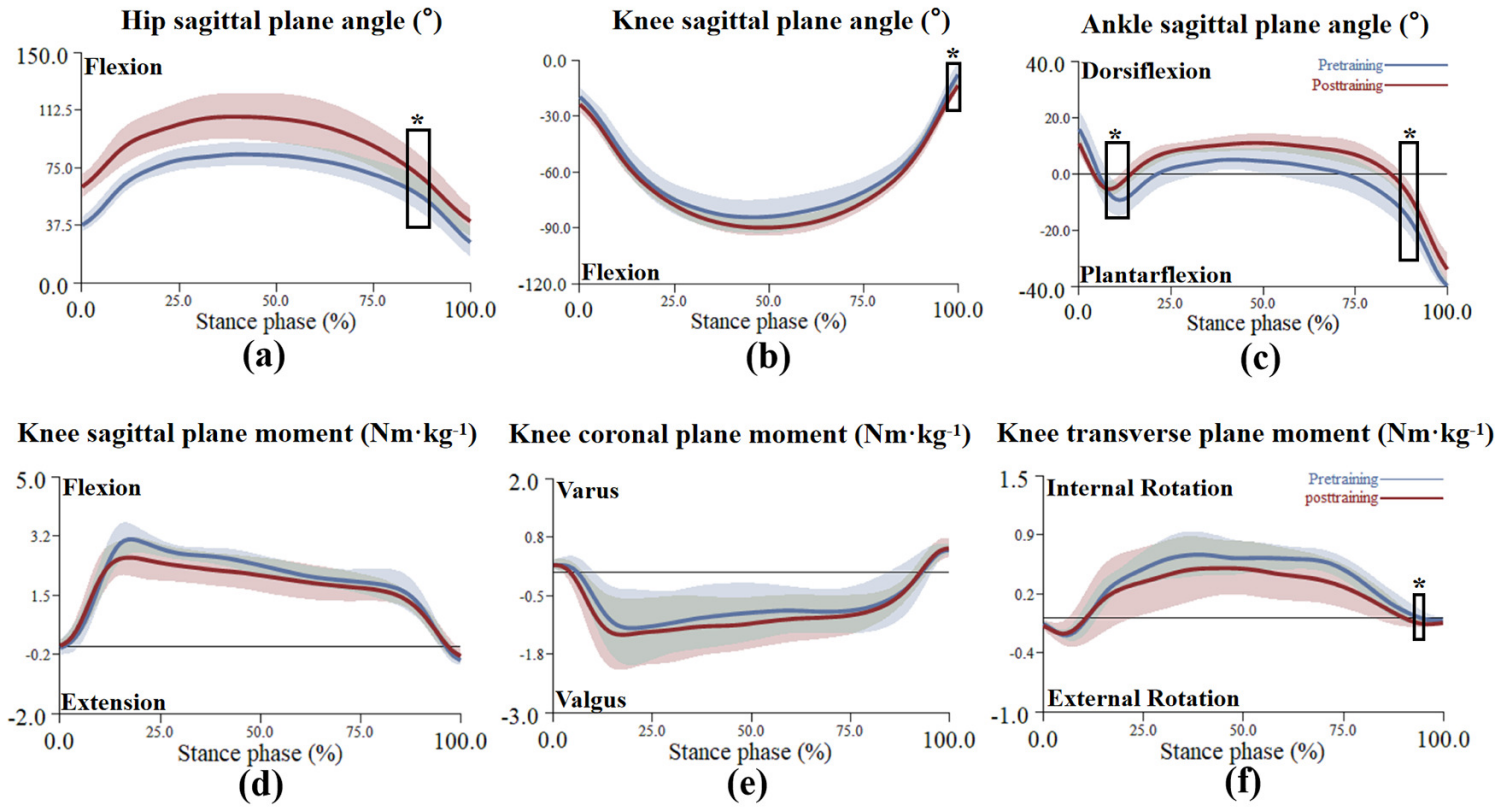
The mean (± SD) value waveform of the resistance training group's lower limb joint angle and knee moment during the stance phase (*indicates significance). **(a–c)** Show the joint sagittal plane angle alterations of the resistance training group between pre-training and post-training during the stance phase. **(d–f)** Show the knee moment alterations of the resistance training group between pre-training and post-training during the stance phase.

For the joint moment, SPM1D analysis revealed no significant differences in hip, knee, and ankle joint moments before and after complex training. However, in the RT group, a significant difference was observed in the knee joint transverse plane moment. Specifically, the transverse plane knee moment exceeded the critical threshold of 4.01 during the 96%–97% phase (*p* = 0.049), indicating a significantly higher knee external rotation moment in the post-training (see Figure 5).

## Discussion

This study aimed to investigate the effects of an 8-week complex training (CT) and resistance training (RT) intervention on the lower limb biomechanical characteristics of the backhand forward lunge. The results revealed significant differences in braking and recovery phase time, knee and ankle range of motion (ROM) between pre-training and post-training. Furthermore, SPM1D analysis indicated that only the RT group exhibited significant changes in the hip, knee, and ankle sagittal plane angles and the knee transverse plane moment from pre-training to post-training. These findings do not fully support our hypothesis. While both training interventions led to improvements in lunge performance, participants appeared to benefit more from resistance training (RT) than from complex training (CT).

The time factor is crucial in evaluating lunge performance, as it determines a player's ability to execute a shot and return to the base position efficiently (4). This study found that a notable reduction in braking phase time was observed only in the CT group. Both the CT and RT groups exhibited a significant reduction in recovery phase time. The RT group demonstrated a larger effect size (1.02 vs. 0.23). These findings suggest that CT and RT produced different improvements. These adaptations are beneficial for badminton performance, as they enable players to return to the baseline more quickly and be better prepared for their opponent's next shot (3).

In terms of range of motion (ROM), this study found that a significant reduction in knee sagittal plane ROM post-training was observed only in the CT group. Additionally, effect size (ES) analysis indicated that both CT and RT groups demonstrated a moderate response in reducing knee sagittal plane ROM (0.63 vs. 0.62), and a small response was observed in hip sagittal plane ROM reduction in both groups (0.57 vs. 0.4). These findings suggest that both training interventions contributed to reductions in hip and knee sagittal plane ROM, potentially improving movement efficiency. However, CT appeared to be more effective than RT in reducing knee sagittal plane ROM. As shown in Table 2, during the execution of the lunge, the hip, knee, and ankle joints predominantly move in the sagittal plane. A reduction in hip and knee sagittal plane movement may indicate enhanced movement efficiency.

Previous studies have identified smaller hip and knee flexion angles as key indicators of effective lunge performance (3, 4, 25). Specifically, executing a lunge with the knee not extending beyond the toes and minimized hip flexion allows players to return to the base position more quickly (3). Notably, this study did not find a significant reduction in hip and knee flexion angles in either training group. Instead, an increase in hip and knee flexion angles was observed in the RT group after training, contradicting previous research on optimal lunge performance. However, this increase may still indicate reduced joint loading and improved lunge efficiency following RT training. Specifically, SPM1D analysis showed that after RT training, the hip flexion angle (79%–87% stance phase) and knee flexion angle (98%–100% stance phase) significantly increased. This study comprehensively considered ROM and joint angles. Although hip and knee flexion angles increased, there was a decreasing trend in knee and hip sagittal plane ROM. At the late stance phase (98%–100%), the lower limb needs to push back to the initial position. As knee flexion angle decreases and approaches extension, the “screw-home mechanism” is activated, tightening the ACL and joint capsule, which increases knee joint loading (26–28). Overloading the knee joint can lead to injuries affecting the ACL, collateral ligaments, and the meniscus (29–31). Therefore, it can be inferred that the significant increase in knee flexion angle during the 98%–100% stance phase helps reduce knee joint load and injury risk.

In terms ankle, Mei et al. (3) examined lunge differences between national-level athletes and recreational college-level players, reporting that recreational players exhibited smaller ankle eversion and internal rotation movements, suggesting poor landing technique of the dominant leg. In this study, the RT group demonstrated greater ankle transverse ROM post-training, indicating an improvement in the landing technique of the dominant leg. Moreover, previous research has shown that lower limb injuries account for 52.15% of all badminton-related injuries, with ankle injuries being the most prevalent (32). Excessive ankle dorsiflexion–plantarflexion can lead to calf fatigue or Achilles tendon injuries (33, 34). SPM1D analysis in this study revealed a reduction in the ankle plantarflexion angle post-training, specifically during the 13%–21% and 88%–92% stance phases. These findings suggest that CT training had a positive effect on ankle injury prevention.

In summary, this study demonstrated that both complex training (CT) and resistance training (RT) contributed to improvements in backhand forward lunge performance. CT primarily enhanced execution efficiency, while RT not only improved efficiency but also showed potential in reducing the risk of knee and ankle injuries. This effect may be explained by the heavy-load resistance training, which can stimulate positive neural, morphological, cellular, and metabolic adaptations (8, 35). These adaptations, in turn, contribute to enhanced athletic performance and potentially lower injury rates (36). Moreover, CT involves alternating between different loads in successive exercises, aiming to achieve post-activation performance enhancement (PAPE)—a temporary improvement in sports performance following high-intensity exercise (37). The magnitude of PAPE is influenced by various factors (37, 38). Players with well-developed muscle strength and extensive experience in resistance training tend to exhibit a more pronounced PAPE effect while individuals with less training experience might still achieve the PAPE effect, but to a lesser extent (37). It may explain the relatively modest benefits of the eight-week complex training intervention. Specifically, participants had not engaged in plyometric or resistance training for the past six months in this study indicating with less training experience, which likely limited the extent of PAPE and the benefits of CT. These findings suggest that CT may be more suitable as an advanced strategy for athletes who already possess a solid strength and training base (15).

It is important to note that these results are specific to amateur male badminton players. Previous research has shown that the backhand forward lunge is associated with a higher injury risk (5), and lower limb injuries account for 52.15% of total badminton-related injuries (32). Given this context, an 8-week resistance training program appears to be particularly beneficial for this population. It is recommended that amateur male badminton players consider incorporating resistance training into their regular regimen to improve performance and reduce injury risk.

This study has several limitations. First, the participants were amateur male badminton players from a sports university, which limits the generalizability of the findings to elite athletes. Second, the analysis focused solely on the backhand forward lunge, which, although important, does not encompass other lunge types or badminton-specific movements. Third, the sample size of this study was relatively small. Future research should include larger cohorts, incorporate elite athletes and female participants, and explore the effects of complex and resistance training across a broader range of badminton movements. Such studies would provide more comprehensive guidance for injury prevention and training optimization.

## Conclusions

After eight weeks of training, both complex training and resistance training improved the backhand forward lunge performance of amateur male badminton players, albeit to varying extents. Complex training was more effective in reducing braking and recovery phase times and decreasing knee sagittal plane range of motion, contributing to greater lunge execution efficiency. Resistance training led to a shorter recovery time, increased ankle transverse range of motion, greater hip and knee flexion angles, and reduced ankle dorsiflexion angles, which collectively enhanced movement efficiency and may reduce the risk of knee and ankle injuries. The resistance training program appears to be more beneficial for this population. Future research should include elite athletes and investigate a broader range of badminton-specific movements to provide more comprehensive guidance on injury prevention and training optimization.

## Data Availability

The raw data supporting the conclusions of this article will be made available by the authors, without undue reservation.
